# Nitric oxide biosensor uncovers diminished ferrous iron-dependency of cultured cells adapted to physiological oxygen levels

**DOI:** 10.1016/j.redox.2022.102319

**Published:** 2022-04-30

**Authors:** Gulsah Sevimli, Matthew J. Smith, Tuba Akgul Caglar, Şükriye Bilir, Melike Secilmis, Hamza Y. Altun, Esra N. Yigit, Fan Yang, Thomas P. Keeley, Roland Malli, Gürkan Öztürk, Giovanni E. Mann, Emrah Eroglu

**Affiliations:** a Molecular Biology, Genetics and Bioengineering Program, Faculty of Engineering and Natural Sciences, Sabanci University, Istanbul, Turkey; b King's British Heart Foundation Centre of Research Excellence, School of Cardiovascular and Metabolic Medicine & Sciences, Faculty of Life Sciences & Medicine, King's College London, 150 Stamford Street, London, SE1 9NH, UK; c Research Institute for Health Sciences and Technologies (SABITA), Istanbul Medipol University, Istanbul, Turkey; d Department of Biotechnology, Gebze Technical University, Kocaeli, Turkey; e Target Discovery Institute, University of Oxford, Oxford, OX3 7FZ, UK; f Molecular Biology and Biochemistry, Gottfried Schatz Research Center, Medical University of Graz, 8010, Graz, Austria; g BioTechMed Graz, Mozartgasse 12/II, 8010, Graz, Austria; h Physiology Department, International School of Medicine, Istanbul Medipol University, Istanbul, Turkey

**Keywords:** Pericellular oxygen, Hyperoxia, Normoxia, NO bioavailability, geNOps, Ferrous iron, Ferric iron, Hydrogen peroxide, Cell culture, Culture media

## Abstract

Iron is an essential metal for cellular metabolism and signaling, but it has adverse effects in excess. The physiological consequences of iron deficiency are well established, yet the relationship between iron supplementation and pericellular oxygen levels in cultured cells and their downstream effects on metalloproteins has been less explored. This study exploits the metalloprotein geNOps in cultured HEK293T epithelial and EA.hy926 endothelial cells to test the iron-dependency in cells adapted to standard room air (18 kPa O_2_) or physiological normoxia (5 kPa O_2_). *We* show that cells in culture require iron supplementation to activate the metalloprotein geNOps and demonstrate for the first time that cells adapted to physiological normoxia require significantly lower iron compared to cells adapted to hyperoxia. This study establishes an essential role for recapitulating oxygen levels *in vivo* and uncovers a previously unrecognized requirement for ferrous iron supplementation under standard cell culture conditions to achieve geNOps functionality.

## Highlights

• Cultured cells expressing iron-dependent geNOps require ferrous iron supplementation.
• Cellular ferrous iron uptake is affected by pericellular oxygen levels.
• Cells cultured under 5 kPa O_2_ levels require lower iron (II) supplementation.
• geNOps biosensor indirectly report intracellular iron levels.

## Graphical abstract

**Figure:**
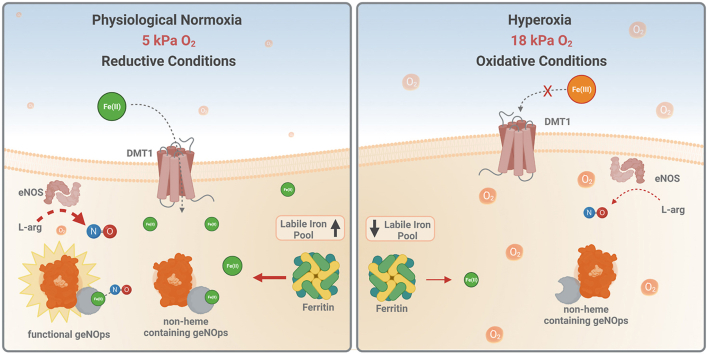

## 1 Introduction

Iron is an essential biometal with versatile biological functions and a key determinant of metalloproteins or enzyme cofactors responsible for electron transfer and catalysis [[1], [2], [3], [4]]. Either lack or excess is detrimental to fundamental metabolism and signal transduction [[5], [6], [7]]. Thus, the regulation of iron metabolism is highly regulated in higher organisms [[8], [9], [10]]. However, delivery of sufficient and non-toxic concentrations of iron to cultured cells and the role of pericellular oxygen remains poorly investigated [11,12].

Supplementation of culture media with animal or human sera containing transferrin is a common strategy to deliver iron to cultured cells [13,14]. Three traditional media are commercially available: supplemented with ferric nitrate, ferrous sulfate, or iron-free [15]. Importantly, ferrous iron is highly soluble at any pH, while oxidized ferric iron at physiological pH without a transport protein or chelator is almost insoluble and poorly bioavailable [16]. Studies suggest that the iron concentration in the extracellular medium *in vivo* ranges within low micromolar levels while ascorbate concentrations can be as high as 1–5 mM [17]. Notably, DMEM contains only 250 nM ferric nitrate and neither ascorbate nor other reducing agents [18]. Moreover, batch and supplier-dependent differences in sera [19] critically affect iron concentration, oxidation, and reduction in cell culture media [20].

Iron and oxygen are intimately linked and are critical cofactors in controlling biochemical pathways to sustain cellular redox balance, such as coordinating ferritin synthesis for iron storage and antioxidant responses [21]. Mammalian cells *in vivo* function under 1–13 kPa O_2_, dependent on the tissue or organ [22] and, in the context of this study, renal epithelial and microvascular endothelial cells usually are exposed to ∼4–6 kPa O_2_ *in vivo* [22]. Notably, cells cultured under standard atmospheric O_2_ (∼18–21 kPa at sea level) are exposed to hyperoxia, i.e., to sustained pro-oxidant stress that significantly alters their redox phenotype [22]. Although iron metabolism in cultured cells is most likely affected by ambient pericellular O_2_ levels, little is known about the relationship between ambient O_2_ and the functionality of metalloproteins in situ.

This study employs the genetically encoded biosensor geNOps [[23], [24], [25]], a metalloprotein consisting of a bacteria-derived non-heme iron containing transcription factor termed GAF-domain that is required to sense intracellular NO dynamics [26]. The GAF domain contains a six-coordinate mononuclear non-heme ferrous iron ion [27]. Electron spin resonance (EPR) spectra of purified proteins treated with NO documented a mononitrosyl complex formation indicating that the GAF domain incorporates one ferrous iron ion [27]. Thus, geNOps only displays full functionality (NO sensitivity) when cells expressing the probe are briefly treated with an iron (II) and ascorbate mixture before an imaging experiment [24,28,29]. Our study provides novel insights into the relationship between ambient O_2_, cellular iron accumulation and intracellular NO bioavailability.

## 2 Material and methods

### 2.1 Chemicals, buffers, and imaging media

Dulbecco's modified Eagle's medium (DMEM), phenol-free DMEM, penicillin and streptomycin, trypsin, and fetal bovine serum (FBS) were purchased from Pan Biotech (Aidenbach, Germany). Cell culture supplements normocin were procured from Invivogen (Toulouse – France), hypoxanthine (HAT) supplements from LGC Standards (Istanbul, Turkey), transfection reagent Polyjet from Signagen (Maryland, USA), d-cysteine from ChemCruz (Heidelberg, Germany), l-Glutathione from AppliChem GmbH (Darmstadt, Germany), Hoechst 33342 from HelloBio (Bristol, UK), ferrozine-reagent from Sigma Aldrich (Taufkirchen, Germany) and FeRhoNox™-1^30^ from Goryo Chemical (Sapporo, Japan). Unless stated otherwise, all other chemicals were purchased from neoFroxx (Einhausen, Germany).

Prior to imaging experiments, cells were incubated for ∼30 min at room temperature in a cell storage buffer consisting of 138 mM NaCl, 2 mM CaCl_2_, 5 mM KCl, 1 mM MgCl_2_, 1 mM HEPES, 2.6 mM NaHCO_3_, 0.44 mM KH_2_PO_4_, 0.34 mM Na_2_HPO_4_, 10 mM d-glucose, 0.1% vitamins, 0.2% essential amino acids, and 1% penicillin/streptomycin, pH 7.4. In live-cell imaging experiments, HEPES-buffered physiological salt solution consisting of 138 mM NaCl, 5 mM KCl, 2 mM CaCl_2_, 1 mM MgCl_2_, 10 mM d-glucose, 10 mM HEPES was prepared. All imaging buffers were adjusted to pH 7.4 using 1 M NaOH.

### 2.2 Cell culture

HEK293T were cultured in complete DMEM, containing 4.5 g/L glucose, 10% FBS, 100 μg/ml streptomycin, and 100 U/ml penicillin, in a humidified incubator (37 °C, 5% CO_2_). EA.hy926 were cultured in complete DMEM containing 4.5 g/L glucose and additional supplements: 2% HAT supplement (consisting of sodium hypoxanthine (5 mM), aminopterin (20 μM), and thymidine (0.8 mM)) and 100 μg/ml normocin. One day before transfection, cells were seeded (∼3–5 x 10^5^ cells per well) on 30 mm glass coverslips No.1 (Glaswarenfabrik Karl Knecht Sondheim, Germany). At ∼80–90% confluency, cells were transfected using PolyJet transfection reagent according to the manufacturer's instructions. All imaging experiments were performed 16–24 h after transfection.

HEK293T and EA.hy926 cells with a phenotype set by long-term culture under room air (18 kPa O_2_, 5 kPa CO_2_) were selected to establish the importance of physiological normoxia (5 kPa O_2_) in NO signaling. Paired cell cultures were maintained under 18 kPa O_2_ or adapted to 5 kPa O_2_ for 5 days in our O_2_-regulated workstations. We have previously demonstrated that cells are able to alter their redox phenotype dependent on the ambient O_2_ level [[31], [32], [33], [34]].

### 2.3 Iron supplementation procedure

Equimolar concentrations (1 mM) of iron compound (FeSO_4_, FeCl_2_, or iron (II) fumarate) and ascorbate in HEPES-buffered physiological salt solution were used to pretreat cells for 20 min at room temperature. Cells were washed twice with PBS to remove excessive iron (II) from cells and incubated in cell storage buffer for 2 h prior to the imaging experiment. Due to the high stability and solubility, FeSO_4_ was used as an iron supplement in all experimental data shown.

### 2.4 Stable cell line generation

A cytosol-targeted O-geNOps (O-geNOp-NES) construct was subcloned into a 3^rd^ generation lentivirus shuttle vector pLenti-MP2 (Addgene #36097) using the following primers: forward 5’-ATAGGATCCGCCACCATGGTGAGTGTG-3' including BamHI restriction site and reverse 5’-ATAGTCGACTTACAAAGTCAATCTTTCT-3' including a stop codon and SalI restriction site. Stable EA.hy926 cell line generation was achieved following optimized protocols as recently described [35]. After positive transduction, cells were cultured for one week in fresh complete DMEM before fluorescence activated cell sorting (FACS). The top 30% of O-geNOp-NES positive cells were selected by detecting red fluorescence emission using an excitation wavelength of 561 nm laser (Filter type: BP 593/40 nm) on a BD Influx Cell Sorter. EA.hy926 cells were sorted, and positively transduced cells were regularly maintained under cell culture conditions before imaging experiments. Stable EA.hy926 cells expressing O-geNOp-NES were seeded on 30 mm glass coverslips one day before an experiment.

### 2.5 Real-time fluorescence imaging and high–resolution confocal microscopy

Real-time cell imaging experiments were performed on inverted widefield epifluorescent microscopes, either an Axio Observer.Z1/7 or Axio Vert.A1 (Zeiss, Germany), equipped with a Plan-Apochromat 20×/0.8 dry objective, a Plan-Apochromat 40×/1.4 DIC (UV) VIR-IR oil immersion objective, and monochrome CCD cameras Axiocam 503. O-geNOp and HyPer7 expressing cells were imaged as described elsewhere [26,35,36].

The intensity and number of cells stained with the labile iron turn-on fluorescent indicator FeRhoNox™-1^30^ were collected and processed using Zen Blue 3.1 software (Zeiss, Germany). Intensity values in regions of interest were divided by cell numbers and normalized for all conditions. To evaluate cell viability, high-resolution fluorescence and phase contrast confocal images were taken using a laser scanning confocal microscope LSM 800 (Zeiss, Germany), equipped with a Plan-Apochromat 20×/0.80 Ph 2 M27 objective. 2048 × 2048 image size pixels were selected, and averaging was set at 4 to obtain high-resolution images. Cells stained with propidium iodide were excited using a 561 nm laser, and respective emission light between 595 and 700 nm was collected using GaAsP-PMT. Hoechst labeled cells were excited using 405 nm laser light, and emission was collected using Multialkali-PMT between 410 and 546 nm. The detector gain, digital detector gain, and pinhole size for all channels were set to 650 V, 1, and 35 μm, respectively. Phase-contrast images were taken using a photodiode detector.

### 2.6 Intracellular iron imaging

Hoechst and FeRhoNox™-1^30^ co-imaging was performed in cells at 70–80% confluency. After cell treatment with FeSO_4_ and/or ascorbate, cells were stained with 5 μg/ml FeRhoNox™-1 in physiological buffer (see above) for 60 min at 37 °C and 5% CO_2_. Subsequently, cells were washed with warm PBS and incubated for 15 min with 10 μg/ml Hoechst at 37 °C and 5% CO_2_. Cells were washed with warm PBS and incubated in a cell storage buffer before imaging.

Perls/Diaminobenzidine (DAB) staining of iron imaging was conducted, as previously described [37]. Cells were first fixed with Karnovsky fixative for 30 min. Cells were imaged on a confocal light microscope LSM800, subsequently further treated with an epoxy embedding procedure to visualize the iron accumulation by electron microscopy. Cells were postfixed with 2% osmium tetroxide (OsO_4_) (Electron Microscopy Sciences, PA, USA) for 30 min at room temperature followed by 2% uranyl acetate (Electron Microscopy Sciences, PA, USA) staining overnight at 4 °C. Following dehydration, cells were embedded in epoxy blocks. Epoxy blocks were trimmed and ultra-sectioned (100 nm) using an ultramicrotome (Leica EM UC7, Leica microsystems, Wetzler, Germany). Thin sections were observed under 3 kV with the BSD detector of the GeminiSEM 500 electron microscope (Carl Zeiss, Jena, Germany).

To visualize intracellular iron accumulation, a scanning electron microscope with energy-dispersive X-ray spectroscopy (SEM/EDX) was used to obtain measurements of iron particles in cells. The same specimens were ultra-sectioned (100 nm) and used for EDX measurements. Measurements were done at 130,000× magnification and 2000 s scanning with 10 kV EHT for carbon and iron atoms. For comparison, iron amounts were normalized to carbon in each selected region of interest.

### 2.7 Cell viability and intracellular glutathione assays

Propidium iodide (PI) and Hoechst staining were applied to identify dead cells and nuclei, respectively, using previously reported protocols [38]. Live cell imaging was performed on a laser scanning confocal microscope LSM 800 (Zeiss, Germany).

EA.hy926 and HEK293T cells were adapted to either 18 or 5 kPa O_2_ for at least five days, reduced glutathione (GSH) extracted using 6.5% trichloroacetic acid (Sigma-Aldrich, UK) and total GSH levels determined using a fluorometric assay [38,39] in a CLARIOstar plate reader (BMG Labtech, Germany) and expressed nmol/mg protein.

### 2.8 Live-cell imaging under controlled oxygen conditions

For experiments under physiological normoxia, cells were cultured in O_2_-regulated workstations (Scitive or InVivo 500, Baker-Ruskinn, U.S.A.) for at least 5 days with O_2_ maintained at 5 kPa O_2_, CO_2_ at 5% and 37 °C [31,32,40,41]. This experimental protocol enable adaptation of the cell redox proteome to a defined pericellular O_2_ level avoiding re-exposure of adapted cells to room air during trypsinization and/or treatments [35,38] Moreover, as shown in Supplementary Fig. 1, adaptation of cells to 5 kPa O_2_ for 5 days avoids stabilization of HIF-1 associated with acute decreases in ambient O_2_ levels [40,41]. An EVOS M7000 imaging system (Thermo Fisher Scientific, USA) was placed in the O_2_-regulated workstations for cell imaging. All imaging experiments were performed at 20× objective magnification (Olympus 20× super-Apochromat) using the 'RFP 2.0' LED light cube (Ex: 542/20 nm Em: 593/40 nm). Cells were washed with PBS and imaged in Earle's Balanced Salt Solution (EBSS) (Sigma-Aldrich, USA) with atmospheric O_2_ set to either 18 or 5 kPa, CO_2_ 5%, and 37 °C. Analysis was performed using ImageJ software. An O_2_-regulated, time-resolved fluorescence plate reader (CLARIOstar, BMG Labtech, Germany) enabled measurements of O-geNOp-NES fluorescence under defined O_2_ conditions (see Fig. 5c and d). Cells adapted to either 18 or 5 kPa O_2_ were seeded into clear bottomed 96-well black plates and cultured for a further 48 h. Cells were pre-incubated with the stated concentrations of FeSO_4_ and ascorbate before being washed with PBS and the addition of EBSS. Cells were incubated for a further hour before being transferred to the plate reader, pre-acclimatized to the correct O_2_ and 5% CO_2_ at 37 °C. Fluorescent readings were taken at 12s intervals at Ex: 532/25 nm and Em: 585/30 nm. Treatments were delivered using the plate reader dual injection system at the stated times and concentrations.

### 2.9 Immunoblotting

EA.hy926 and HEK293T cells were cultured for at least 5 days at 18, 5 and 1 kPa O_2_. Cell lysates were collected with SDS lysis buffer containing protease inhibitors on ice, before protein separation by SDS-PAGE and transfer to polyvinylidene difluoride membrane. The membranes were probed with primary antibodies for HIF1α (BD Biosciences, USA, 610959), eNOS/NOS3 (Santa Cruz, USA, sc376751), FTH1 (Cell Signaling Technology, USA, 3998), and β-actin (Sigma-Aldrich, USA, A1978), before addition of the appropriate HRP-conjugated secondary antibody. Protein expression was determined by chemiluminescence with images captured using a gel documentation system (G-BOX, Syngene Ingenius Bioimaging) and densitometry analysis using Image J software (NIH, USA).

### 2.10 Statistical analysis

All acquired imaging data were analyzed using GraphPad Prism software (GraphPad Software, San Diego, CA, USA). All experiments were repeated at least three times in different cell cultures. The number of experiments is given as 'N', and the total number of cells imaged are indicated as 'n.' For instance: 3/18 indicates N = 3 (triplicate cultures) and n = 18 (number of cells imaged in this experiment). Unless stated otherwise, all statistical data are presented as mean ± SD in addition to the representative real-time traces shown as curves. geNOps signals are based on fluorescence quenching, and for convenient representation, biosensor responses have been were inverted as initially described elsewhere [23,24,26,42] using the formula *ΔF*_*Intensity*_ = *(1-(F/F*_*0*_)*100). For relative comparison of two or more groups in a given experiment, the maximum signal was calculated as 100%, and all other groups were normalized, respectively. Statistical comparison of two groups was evaluated using a two-tailed Student *t*-test. Statistical comparisons of multiple groups one-way ANOVA analyses of variances with post-test Dunnett (comparison of all pairs of columns to control) were performed. All single values for concentration-responses were performed at least in triplicate.

## 3 Results

### 3.1 Treatment with iron(II) and ascorbate is essential for geNOps functionality

**Fig. 1:**
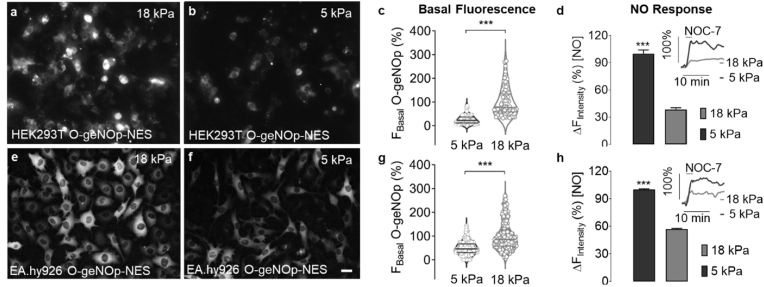
Effects of ambient oxygen levels on basal and NOC-7 induced geNOps responses in HEK293T and EA.hy926 cells. Representative widefield images of HEK293T cells stably expressing O-geNOp-NES adapted for five days to a, hyperoxic (18 kPa) or b, normoxic (5 kPa) O_2_ levels. Scale bar represents 20 μm c, Violin plot shows the basal fluorescence intensity of O-geNOp-NES in HEK293T cells after adaptation to 18 kPa (light grey dots, n = 3/70) or 5 kPa (dark grey dots, n = 3/73) O_2_. Average of cells adapted to 18 kPa O_2_ have been set as 100% and average of cell adapted to 5 kPa O_2_ have been normalized, respectively. d, Bar and inset show, respectively, maximum O-geNOp-NES response and representative real-time traces of NO in cells in response to 10 μM NOC-7 after adaptation to 18 kPa (light grey bar and curve, n = 3/33) or 5 kPa O_2_ (dark grey bar and curve, n = 3/34). e-h, Same experimental setup in EA.hy926 cells as shown in the panels a–d. g, Violin plot shows the measurements of basal fluorescence intensity in EA.hy926 cells after adaptation to 18 kPa (light grey plot, n = 3/327) or 5 kPa (dark grey plot, n = 3/322) O_2_. h, Bars indicate maximum O-geNOp-NES signals in response to 10 μM NOC-7 in EA.hy926 cells adapted to 18 kPa (light grey bar, n = 3/40) or 5 kPa (dark grey bar, n = 3/41) O_2_. Insets show representative real-time traces of cells expressing O-geNOp-NES adapted to 18 kPa or 5 kPa O_2_ in response to 10 μM NOC-7. Students *t*-test was applied for statistical analysis. All values denote mean ± S.D., ***P < 0.0001.

Standard cell culture settings under room air conditions (18 kPa O_2_) are unphysiological for most cell types; thus, we examined the effects of physiological O_2_ levels (5 kPa) in HEK293T and EA.hy926 cell lines stably expressing the orange variant of geNOps. Both EA.hy926 and HEK293T cells only showed a stabilization of HIF-1⍺ at 1 kPa O_2_, indicating that long-term culture of cells under 5 kPa does not induce a hypoxia phenotype (Supplementary Figs. 1a and d). Both cell lines adapted for five days to 18 kPa O_2_ levels showed robust geNOps expression, while cells adapted to 5 kPa O_2_ displayed significantly lower basal fluorescence intensity indicating a delayed chromophore maturation (Fig. 1a–c & e-g). Nevertheless, NOC-7, a potent NO donor [23], evoked a robust geNOps signal in both cell lines adapted to physiological normoxia. In contrast, in cells adapted to standard culture under hyperoxia (18 kPa O_2_), geNOps displayed marginal changes in response to the potent NO donor (Fig. 1d,h) in a concentration dependent manner (Supplementary Fig. 2) Overall, these observations establish a critical role for physiological pericellular O_2_ in NO bioimaging, yet it remains unclear whether ambient O_2_ concentrations affect NO bioavailability [43] or cellular iron (II) uptake and thereby geNOps activity.

Short-term (24 h) or long-term (21 days) culture of HEK293T cells under 18 kPa O_2_ in different commercially available media such as Dulbecco's Minimal Essential Media (DMEM), Advanced DMEM, F12, and F12K containing ferrous iron, ferric iron, or ascorbate, respectively, only led to marginal activation of geNOps (Supplementary Fig. 3). However, geNOps displayed full functionality upon treatment of cells with 1 mM FeSO_4_ and 1 mM ascorbate for 20 min prior to imaging experiments (Supplementary Fig. 3). Long-term (14 days) adaptation of cells to sub-toxic concentrations of ascorbate (ranging from 12 to 96 μM) or FeSO_4_ (ranging from 7 to 56 μM) in DMEM also failed to activate geNOps (Supplementary Fig. 4). Overall, these data suggest that commercially available cell culture media require iron (II) and ascorbate supplementation for metalloprotein functionality.

### 3.2 Imaging intracellular distribution of ferric and ferrous iron

Our results so far demonstrate the requirement for iron (II) supplementation to cells to achieve full functionality of geNOps, yet the spatial distribution and quantification of the labile iron pool remain unclear. We initially performed high-resolution confocal imaging experiments using the fluorescent indicator FeRhoNox-1, a specific probe to detect cytosolic labile iron [30]. Collecting multiple focal planes in the Z-direction in HEK293T cells adapted to 18 kPa O_2_ cells pretreated with FeSO_4_ and ascorbate revealed that a significant amount of the probe was also detectable on the surface of cells (Supplementary Fig. 5a). To further confirm whether iron (II) accumulates on the cell surface, we imaged cells stained with Perls and 3’-diaminobenzidine (DAB). Cells treated with ascorbate only were comparable to the control group. However, cells treated with FeSO_4_ in the absence of ascorbate displayed a remarkable accumulation of extracellular iron particles, indicating that most supplemented iron precipitates without entering the cells and aggregates on the surface of the cell membrane (Supplementary Fig. 5b).

In contrast, FeSO_4_ and ascorbate co-treatment led to negligible iron accumulation on the cell surface, whereas intracellular aggregates were detectable (Supplementary Fig. 5b). These results confirm that a strong reducing agent is essential to keep iron in solution reduced under hyperoxic conditions, allowing cells to internalize soluble iron from the culture media. FeRhoNox-1 imaging in cells adapted to either 5 or 18 kPa O_2_ and treated with FeSO_4_ only or in combination with ascorbate displayed higher levels of intracellular labile iron under physiological O_2_ conditions (Supplementary Figs. 5c and d), demonstrating the critical role of the redox state of iron in cellular uptake.

**Fig. 2:**
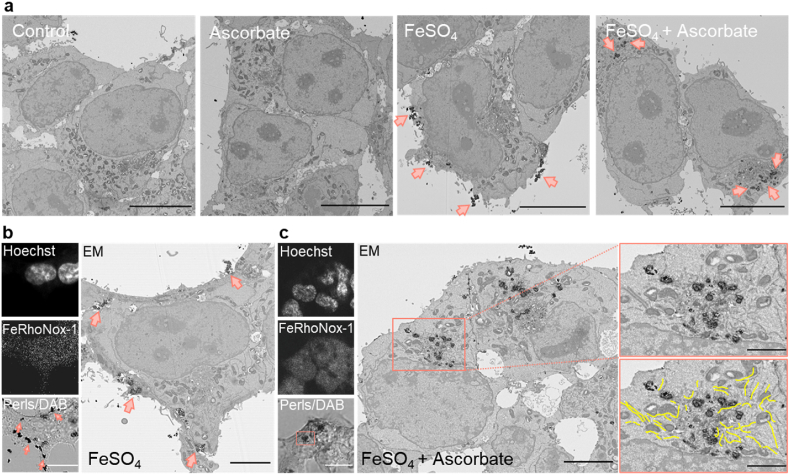
geNOps functionality correlates with cellular iron (II) uptake. a, Representative electron micrographs of HEK293T cells stained with Perls/DAB under control condition (1^st^ image), treated with 1 mM ascorbate (2^nd^ image), treated with 1 mM FeSO_4_ (3^rd^ image), or treated with 1 mM FeSO_4_ + 1 mM ascorbate (4^th^ image). Pink arrows indicate accumulated iron particles. Scale bars indicate 10 μm b, Representative low-magnification images of HEK293T cells stained with Hoechst, FeRhoNox-1, and Perls/DAB for CLEM experiments upon treatment with 1 mM FeSO_4_ for 20 min c, Representative CLEM images of HEK293T cells following treatment with 1 mM ascorbate +1 mM FeSO_4_ for 20 min. Micrographs (pink bordered) show high magnification of the indicated region. The yellow lines indicate structures of the endoplasmic reticulum (ER). Scale bars of light microscopy images represent 15 μm, 5 μm for low magnification, and 2 μm for high magnification EM images. Representative data were selected from n = 8–15 replicates. (For interpretation of the references to color in this figure legend, the reader is referred to the Web version of this article.)

These results were further validated by electron microscopy (EM) (Fig. 2a). Correlative light and electron microscopy (CLEM) experiments confirmed the accumulation of extra- and intracellular iron. FeRhoNox-1 stained cells were less suitable for this protocol, as the chemical dye targets undefinable intracellular regions. Thus, we exploited Perls stained cells, intensified with diaminobenzidine (DAB), and then correlated high-resolution bright-field and EM images of the same cells (Fig. 2b and c). Our CLEM approach confirmed that cells treated with FeSO_4_ only displayed visible dark punctae, indicating the precipitation and accumulation of iron on the cell surface (Fig. 2b, left panel). These data correlate with EM images, confirming that ascorbate is required to internalize extracellular iron (Fig. 2b, right panel). Notably, there was an apparent accumulation around the endoplasmic reticulum (ER) without signs of ER stress (Fig. 2c, right panel).

### 3.3 Optimization of the iron-supplementation procedure

**Fig. 3:**
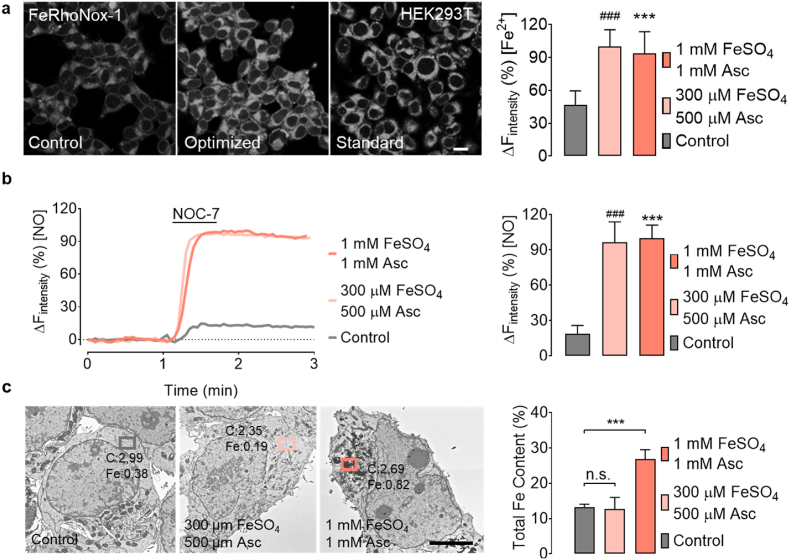
Optimization of iron (II) supplementation in HEK293T cells adapted to 18 kPa O_2_. a, Representative confocal images of HEK293T cells stained with FeRhoNox-1 under non-treated conditions (left image), treated with optimized iron (II) levels of 300 μM FeSO_4_ + 500 μM ascorbate for 15 min (middle image) and standard procedure 1 mM FeSO_4_ + 1 mM ascorbate for 20 min (right image). Bars show FeRhoNox-1 intensities under control conditions (grey bar, n = 7/443), optimized iron (II) concentration conditions (light pink bar, n = 10/469), or standard iron (II) treatment conditions (pink bar, n = 11/511). b, Representative real-time traces of geNOps signals in HEK293T cells in response to 10 μM NOC-7 (NO donor). Representative curves show NO signals in cells without iron (II) treatment (grey curve), with optimized iron (II) solution (light pink curve) and cells treated with the standard iron protocol (pink curve). Bars show respective maximum NO responses: control conditions; grey bar, n = 3/36, optimized iron supplementation; light pink bar, n = 3/48, and standard conditions; pink bar, n = 3/53. c, Representative micrographs of SEM/EDX measurements in HEK293T cells untreated (left image), optimized iron supplementation (middle image), or treated with the standard iron protocol (right image). Bars represent total cellular iron content without (grey bar, n = 3) or upon optimized (light pink bar, n = 3) or standard iron (II) supplementation (pink bar, n = 3). Dunnett's Multiple Comparison Test was applied to compare the total iron content following treatments relative to the control column. All values denote mean ± S.D., *P < 0.0001* (^###^Control vs 300 μM FeSO_4_ + 500 μM ascorbate; ***Control vs 1 mM FeSO_4_ + 1 mM ascorbate). Scale bar represents 5 μm. (For interpretation of the references to color in this figure legend, the reader is referred to the Web version of this article.)

We next sought to optimize acute iron (II) supplementation by lowering the reducing agent and iron compound concentrations. Employing Taguchi guidelines [44], we optimized all three parameters: FeSO_4_ and ascorbate concentrations and the incubation time (Supplementary Table 1). This approach established that a minimum concentration of iron (II) compound, reducing agent, and incubation time of 300 μM, 500 μM, and 15 min, respectively, is required to activate geNOps biosensor (Supplementary Fig. 6). As shown in Fig. 3a, the optimized iron supplementation protocol resulted in FeRhoNox-1 signals similar to those achieved by our initial iron loading protocol (1 mM FeSO_4_ and 1 mM ascorbate for 20 min). These results show that lower levels of FeSO_4_ and ascorbate provision under room air conditions yield similar levels of intracellular labile iron documented by geNOps functionality and FeRhoNox-1 (Fig. 3b).

Importantly, scanning electron microscopy with energy-dispersive X-ray spectroscopy (SEM/EDX) analysis for elemental identification and quantitative compositional information confirmed that optimized iron (II) concentrations did not lead to accumulation of intracellular iron (II) in undefinable structures (Fig. 3c, and Supplementary Fig. 7). Overall, our findings suggest that lower FeSO_4_ and ascorbate concentrations are sufficient and necessary to activate metalloprotein geNOps under standard cell culture conditions (18 kPa O_2_).

**Fig. 4:**
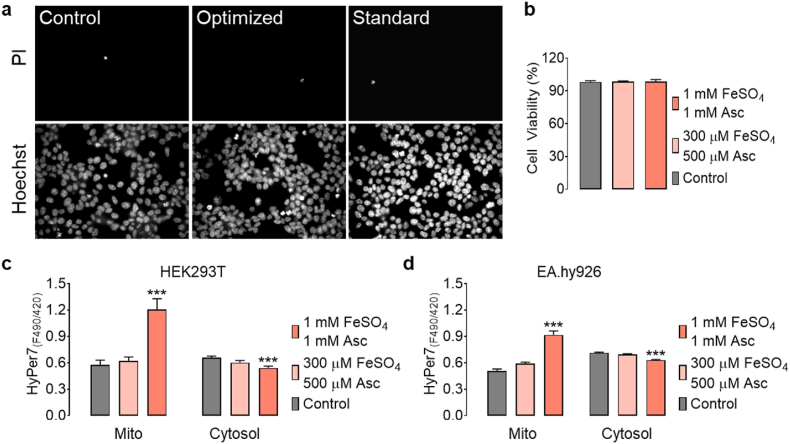
Analysis of cell toxicity and mitochondrial reactive oxygen species following iron (II) supplementation in HEK293T and/or EA.hy926 cells adapted to 18 kPa O_2_. a, Representative widefield images of HEK293T cells co-stained with propidium iodide and Hoechst under control conditions (left images), treated with 300 μM FeSO_4_ + 500 μM ascorbate for 15 min (middle images), or treated with 1 mM FeSO_4_ + 1 mM ascorbate for 20 min (right images). b, Bars represent cell viability under control conditions (grey bar, n = 6/60), 300 μM FeSO_4_ + 500 μM ascorbate (light pink bar, n = 6/60), and 1 mM FeSO_4_ + 1 mM ascorbate (pink bar, n = 6/60). (c) Bars show basal HyPer7 ratio levels in the mitochondria and cytosol of HEK293T cells under control conditions (mito: grey bar, n = 28/280; cyto: n = 30/300) and following acute treatment with 300 μM FeSO_4_ + 500 μM ascorbate (mito: light pink bar, n = 27/270; cyto: n = 30/300), or 1 mM FeSO_4_ + 1 mM ascorbate (mito: pink bar, n = 25/250; cyto: n = 30/300). d, Basal HyPer7 ratio levels in mitochondria and cytosol of EA.hy926 cells under control conditions (mito: grey bar, n = 18/180; cyto: n = 34/340) and following acute treatment with 300 μM FeSO_4_ + 500 μM ascorbate (mito: light pink bar; n = 18/180; cyto: n = 34/340), or 1 mM FeSO_4_ + 1 mM ascorbate (mito: pink bar, n = 18/180; cyto: n = 34/340). HyPer7 ratio signals have been calculated by post image processing from static images (F_490_/F_420_). Dunnett's Multiple Comparison Test was applied to compare all treatments relative to the control column. All values denote mean ± S.E.M., *P < 0.0001.* (For interpretation of the references to color in this figure legend, the reader is referred to the Web version of this article.)

Cell viability and mitochondrial reactive oxygen species (ROS) generation were examined following acute iron (II) supplementation in HEK293T cells under 18 kPa O_2_. Cell viability and mitochondrial ROS levels remained unaffected by iron (II) supplementation (Fig. 4). However, treatment of HEK293T and EA.hy926 cells with 1 mM FeSO_4_ and 1 mM ascorbate led to robust increases in mitochondrial H_2_O_2_ levels and significant decreases in the cell cytosol measured using the ultrasensitive H_2_O_2_ biosensor HyPer7 [35,45] (Fig. 4c and d). Significantly, optimized iron (II) concentrations did not increase mitochondrial H_2_O_2_ levels 24 h after iron (II) supplementation in both cell lines, however, only in HEK293T cells the cytosolic H_2_O_2_ levels were reduced (Fig. 4c and d). Basal intracellular GSH levels were lower in EA.hy926, but not in HEK293T cells adapted to 5 kPa than cells adapted to 18 kPa O_2_, consistent with diminished oxidative stress in cells cultured under physiological normoxia [36] (Supplementary Fig. 8a). Moreover, basal mitochondrial H_2_O_2_ levels in EA.hy926 cells were comparable under 18 kPa and 5 kPa, suggesting that mitochondrial ROS levels were affected negligibly by physiological normoxia (Supplementary Fig. 8c).

**Fig. 5:**
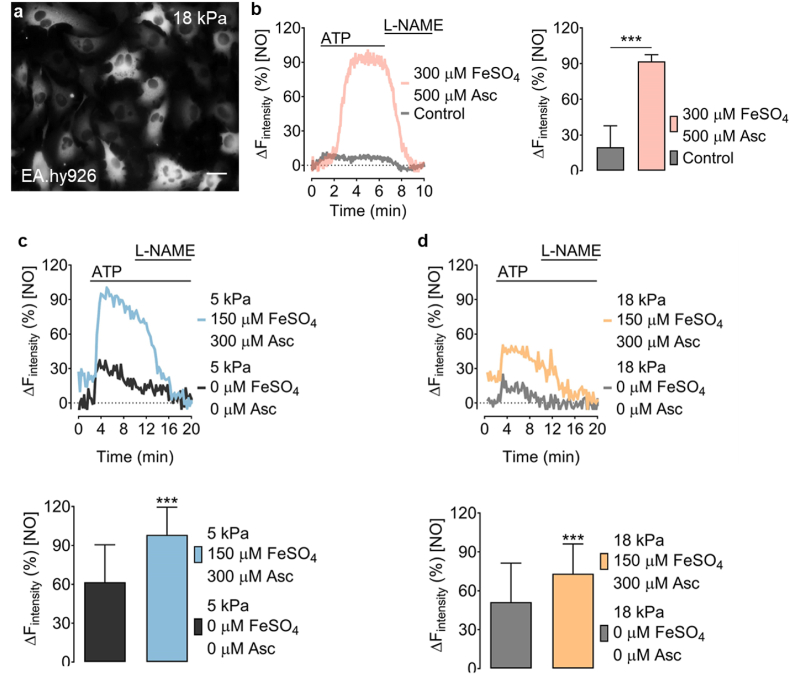
Visualizing intracellular NO signals in endothelial cells adapted to physiological normoxia or hyperoxia. a, Representative widefield image of EA.hy926 cells stably expressing O-geNOp-NES adapted to 18 kPa O_2._ b, Representative real-time traces of endogenous NO signals in EA.hy926 cells in response to 100 μM ATP and subsequent administration of 500 μM l-NAME under control conditions (no iron treatment, grey curve, and bar, n = 3/54) or pretreated with the optimized iron solution (pink curve and bar, n = 3/29). c, Representative real-time traces show endogenous NO signals in EA.hy926 cells, expressing O-geNOp-NES adapted to 5 kPa O_2_. Cells were treated with 30 μM ATP and subsequently with 1 mM l-NAME. The blue curve shows cells pretreated with 150 μM FeSO_4_ + 300 μM ascorbate and the dark grey curve the NO response in cells without any treatment prior to imaging. Bars show maximum NO responses in cells without iron (II) treatment (dark grey bar, n = 3/107) or treatment with 150 μM FeSO_4_ + 300 μM ascorbate (blue bar, n = 3/107). d, Experiments were conducted with cells under the same treatment conditions shown in panel (c), but following adaptation of cells for five days to 18 kPa O_2_. Light grey curve and bars show NO responses with no iron (II) treatment (n = 3/107), and yellow curve and bars show NO responses in cells treated with 150 μM FeSO_4_ + 300 μM ascorbate (n = 3/107). Student's *t*-test, was performed to determine statistical differences. All values denote mean ± S.D., ***P < 0.0001. (For interpretation of the references to color in this figure legend, the reader is referred to the Web version of this article.)

### 3.4 The role of ambient oxygen levels on geNOps functionality in endothelial cells

We next sought to examine the effects of optimized iron (II) supplementation in EA.hy926 endothelial cells capable of generating intracellular NO in response to the GPCR agonist adenosine triphosphate (ATP), which robustly triggers intracellular calcium mobilization to activate eNOS [46] (Fig. 5a). Treatment of endothelial cells adapted to 18 kPa O_2_ with ATP caused a robust intracellular geNOps signal (inhibitable by nitro-l-arginine methyl ester) in cells pretreated with the optimized iron (II) and ascorbate concentration. In contrast, the geNOps signal in non-iron treated cells was negligible (Fig. 5b). Our results indicate that treating endothelial cells with iron (II) in combination with ascorbate is necessary for activating geNOps under standard hyperoxic culture conditions. We hypothesized that the iron (II) concentration could be lowered further by adapting endothelial cells to physiological normoxia (5 kPa O_2_) to mimic O_2_ levels *in vivo* [22,41]. EA.hy926 cells stably expressing O-geNOp-NES were adapted to either 18 or 5 kPa O_2_ for at least five days. Of note, eNOS protein expression is unaltered by differences in oxygen culture conditions in EA.hy926 cells (Supplementary Fig. 1c).

We initially treated cells adapted to 5 kPa O_2_ with even lower iron (II) (150 μM FeSO_4_ and 300 μM ascorbate). ATP stimulated NO production in these cells induced a robust geNOps signal that was diminished upon subsequent addition of the NO synthase inhibitor l-NAME (Fig. 5c). These results confirm our hypothesis that culturing endothelial cells under physiological normoxia requires treatment with significantly lower iron (II) and ascorbate concentrations. However, when EA.hy926 cells were adapted to standard cell culture hyperoxia (18 kPa O_2_) and pretreated with the same concentrations of iron (II) and ascorbate (150 μM FeSO_4_ and 300 μM ascorbate), the geNOps signal in response to ATP was significantly decreased (Fig. 5d). Analysis of the ferritin heavy chain protein (FTH1) expression in EA.hy926 cells showed a significant increase in ferritin under hyperoxic conditions, potentially providing a larger cellular capacity to sequester labile iron and therefore leave it unavailable for O-geNOp incorporation and function (Supplementary Figs. 1b and e).

## 4 Discussion

The present study, investigating live-cell NO imaging in HEK293T and EA.hy926 cells, provides direct evidence that ambient O_2_ levels during cell culture critically affect: (i) ferrous iron accumulation, (ii) ferrous iron dependent geNOps responses, and (iii) intracellular NO bioavailability. These findings highlight the importance of recapitulating oxygen levels encountered by cells and tissues *in vivo* and the necessity of iron (II) supplementation in cells cultured under physiological normoxia or standard hyperoxic culture conditions.

We employed a genetically encoded NO biosensor geNOps as a model system for a non-heme iron (II) containing metalloprotein in cultured cells [23]. A previous study demonstrated that geNOps functionality requires ferrous iron supplementation for optimal live-imaging of changes in intracellular NO levels [29]. We extended this experimental approach to probe the role of iron (II) supplementation and ambient oxygen levels on the functionality of the geNOps biosensor by exploiting the NO-sensitivity as a direct read-out for the probe's iron-dependent (dys)functionality.

Cells chronically exposed to hyperoxic O_2_ levels (18 kPa O_2_) showed a robust geNOps expression, yet the biosensor lacked sensitivity for exogenous NO administration (Fig. 1). Cells adapted to physiological normoxia (5 kPa O_2_) displayed lower basal fluorescence in both HEK293T and EA.hy926 cells, as expected due to the requirement for O_2_ in the maturation of the fluorophore [47]. A recent study investigated chromophore maturation under certain oxygen conditions including 9, 12, 15, and 21 kPa O_2_ with differently colored purified pre-mature FPs. The authors document that green FPs maturation was O_2_-independent while red-shifted FPs showed significant maturation delay under lower O_2_ conditions [48] in line with our observations. However, to our surprise, geNOps functionality was significantly improved under physiological O_2_ levels (Fig. 1, Fig. 5), raising questions about whether NO bioavailability increased due to reduced scavenging of NO [43] or whether iron (II) dependent biosensor activity was enhanced under physiological normoxia. In the context of the latter question, two well-established pathways regulate iron internalization in cultured cells: (i) ferric iron complex with serum protein transferrin and (ii) iron uptake via a divalent metal transporter (DMT1) [49], or subtypes of transient receptor potential (TRP) channels [50].

Notably, in cells adapted to different commercially available culture media containing ferric and/or ferrous iron supplemented with transferrin, the NO donor NOC-7 failed to activate geNOps functionality, probe sensitivity was recovered following brief treatment with fresh FeSO_4_ and/or ascorbate (Supplementary Fig. 3). These observations strongly suggest the oxidation of ascorbate and ferrous iron under room air conditions. We demonstrated that cell treatment with supraphysiological concentrations of ascorbate (but not GSH and cysteine (data not shown)) and ferrous iron for 20 min significantly enhanced the geNOps signal in cells adapted to 18 kPa O_2_. Interestingly, cell treatment with ascorbate alone only improved geNOps functionality marginally (Supplementary Fig. 3). This observation is critical and in line with previous reports [51]. *In vitro* studies show that different reducing agents, including flavin mononucleotide, ascorbate, sodium dithionite, and superoxide, cause reduction of the ferritin iron core and cause iron release [51]. The increased labile iron is available for iron (II)-chelating agents or proteins like geNOps.

In contrast, other studies show that reductants such as glutathione poorly reduce ferritin [52] and are less able to mobilize free iron, underpinning our findings that the reductants GSH and d-cysteine are less able to increase geNOps functionality (data not shown). Our results demonstrate that pretreatment of cells with ascorbate is suitable but not sufficient to gain geNOps functionality (Supplementary Fig. 3). These observations underpin the hypothesis that only a strong reducing agent can mobilize intracellular iron, as documented by the increase in geNOps signal (Supplementary Fig. 3). However, additional provision of ferrous iron further maximizes the functionality of metalloproteins. Thus, a reductive cytosolic and extracellular environment and iron (II) supplementation in culture media are essential to activate the non-heme iron containing metalloprotein geNOps fully.

Significantly, the FeRhoNox-1 signal in cells adapted to physiological normoxia (5 kPa) displayed the same levels of labile iron compared to cells adapted to hyperoxia (18 kPa) (Supplementary Figs. 5c and d). These findings may indicate that the cytosolic reductive/oxidative environment remains unaffected by pericellular oxygen levels, as evidenced in HEK293T cells, in which similar GSH levels were measured in cells adapted to 18 or 5 kPa O_2_ (Supplementary Fig. 8). Although both approaches (geNOps and FeRhoNox-1) document efficient iron (II) uptake following brief cell treatment with ferrous iron and ascorbate, the intracellular distribution of iron remained elusive. Accumulation of the FeRhoNox-1 probe to undefinable intracellular regions precluded us from identifying the labile iron pool with a cellular compartment. Correlative light and electron microscopy indeed showed that cell treatment with iron (II) only led to accumulation on the cell surface (Fig. 2). In contrast, in the presence of ascorbate, iron (II) no longer accumulated on the cell surface and appeared enclosed within undefinable structures surrounding the endoplasmic reticulum, presumably particles of excessive iron precipitated in lysosomal structures to be recycled. Importantly, treatment of cells with significantly lower iron (II) and ascorbate concentrations no longer caused intracellular accumulation of excessive iron particles, as evidenced by scanning EM and energy-dispersive X-ray spectroscopy (Fig. 3).

Although increases in the labile iron pool may have toxic effects due to the ability of iron to generate hydroxide (OH^−^) and hydroxyl radical (Fenton Reaction) [53], imaging mitochondrial H_2_O_2_ levels confirmed that optimized iron (II) treatment (300 μM FeSO_4_, 500 μM ascorbate for 15 min) did not increase H_2_O_2_ generation in cells transfected with HyPer7 (Fig. 4). Importantly, utilizing EA.hy926 cells stably expressing mitochondria-targeted HyPer7, we established that basal H_2_O_2_ levels were similar in cells adapted to 18 or 5 kPa O_2_ (Supplementary Fig. 8c). These observations are critical, as it has been reported that cells shift their metabolism to reduced aerobic oxidative respiration under oxygen-poor conditions with a decreased electron transport rate, leading to collapse of mitochondrial membrane potential (Δ*ψ*_*m*_) probably due to increased levels of mitochondrial ROS and enhanced NO formation [43,54]. Notably, cells adapted to physiological normoxia (5 kPa O_2_) required significantly lower iron (II) and ascorbate concentrations to achieve geNOps signals comparable to those measured in EA.hy926 cells adapted to standard room air culture conditions (Fig. 5).

In conclusion, cultured cells expressing the metalloprotein geNOps displayed marginal functionality when cultured under standard room air conditions, whilst geNOps sensitivity was enhanced following long-term culture under physiological normoxia. Our results further demonstrate that optimized iron (II) supplementation *in vitro* is required to achieve maximal functionality of geNOps and, most likely other metal-containing proteins. Critically, our study raises an important caveat about interpreting biochemical activation and functionality of metal-containing enzymes in cells cultured under standard room air conditions. Given the caveats concerning chemical fluorescence probes [55], further advances in novel genetic biosensors for high-resolution, real-time imaging of labile iron levels are warranted to enhance our understanding of iron-dependent metabolism and signaling pathways in cells under well-defined physiological oxygen levels [56].

## Author contributions

G.E.M. and E.E. conceptualized the study; G.S., M.J.S., T.A.C., and Ş.B. developed the methodology; G.S., M.J.S., H.Y.A., M.S., T.A.C., Ş.B., F.Y., and E.N.Y. performed and analyzed the experiments; H.Y.A. and M.S. generated stable cell lines; G.S., T.A.C., M.J.S., G.E.M., and E.E. wrote the manuscript, which all authors reviewed. R.M. and G.Ö. provided reagents, equipment, and protocols. G.E.M. and E.E. are guarantors of this study, with responsibility for the integrity of the data and data analysis.

## Declaration of competing interest

The authors declare that they have no known competing financial interests or personal relationships that could have appeared to influence the work reported in this paper.
