# Supplementary material for: Nitric oxide biosensor uncovers diminished ferrous iron-dependency of cultured cells adapted to physiological oxygen levels
Source: Redox Biol. 2022 Apr 30;53:102319. doi: 10.1016/j.redox.2022.102319 (PMC9079701; doi:10.1016/j.redox.2022.102319)

**Nitric oxide biosensor uncovers diminished ferrous iron-dependency of cultured cells adapted to physiological oxygen**

*Gulsah Sevimli^1,#^, Matthew J. Smith^2,#^, Tuba Akgul Caglar^1,3,#^, Şükriye Bilir^3,#^, Melike Secilmis^1^, Hamza Y. Altun^1,^, Esra N. Yigit^3,4^, Fan Yang^2^, Thomas P. Keeley^5^, Roland Malli^6,7^, Gürkan Öztürk^3,8^, Giovanni E. Mann^2,¶,^* and Emrah Eroglu^1,3,¶,^**

^1^Molecular Biology, Genetics and Bioengineering Program, Faculty of Engineering and Natural Sciences, Sabanci University, Istanbul, Turkey

^2^King's British Heart Foundation Centre of Research Excellence, School of Cardiovascular and Metabolic Medicine & Sciences, Faculty of Life Sciences & Medicine, King's College London, 150 Stamford Street, London SE1 9NH, U.K.

^3^Research Institute for Health Sciences and Technologies (SABITA), Istanbul Medipol University, Istanbul, Turkey

^4^Department of Biotechnology, Gebze Technical University, Kocaeli, Turkey

^5^ Target Discovery Institute, University of Oxford, Oxford OX3 7FZ, U.K.

^6^Molecular Biology and Biochemistry, Gottfried Schatz Research Center, Medical University of Graz, 8010 Graz, Austria

^7^BioTechMed Graz, Mozartgasse 12/II, 8010 Graz, Austria

^8^Physiology Department, International School of Medicine, Istanbul Medipol University, Istanbul, Turkey

^#^Authors contributed equally

^¶^Authors coordinated the study

**Correspondence:** [giovanni.mann@kcl.ac.uk](mailto:giovanni.mann@kcl.ac.uk) and [emrah.eroglu@sabanciuniv.edu](mailto:emrah.eroglu@sabanciuniv.edu)

Supplementary Figures 1-8

Supplementary Table 1

**Supplementary Figures**


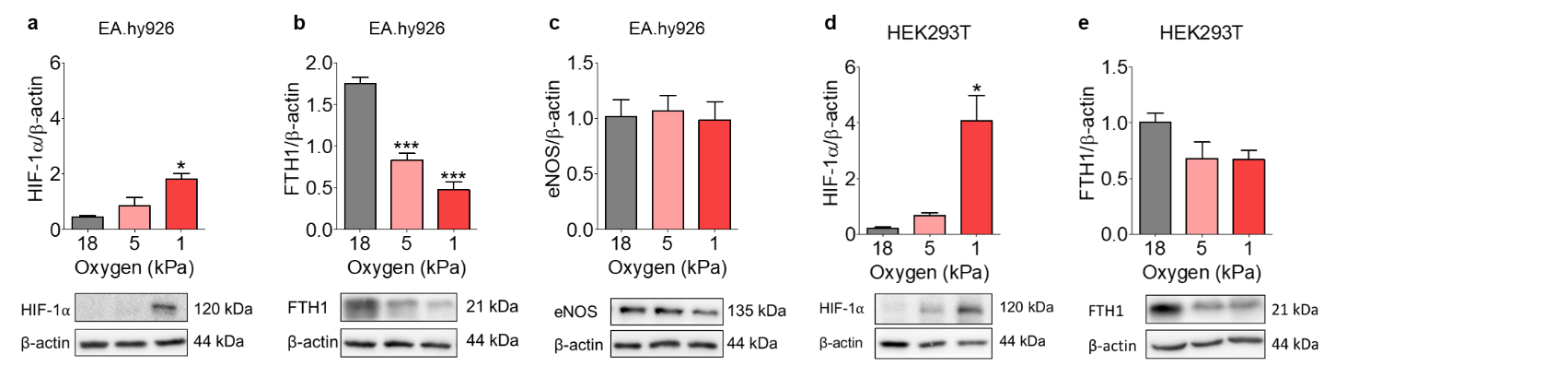


**Supplementary Fig. 1: Protein expression of hypoxia-inducible factor 1-alpha, endothelial nitric oxide synthase and the ferritin heavy chain in EA.hy926 and HEK293T cells cultured long-term under hyperoxia, normoxia or hypoxia.** EA.hy926 and HEK293T cells were cultured at 18, 5 and 1 kPa O_2_ for at least 5 days and lysates were immunoblotted for protein expression of HIF-1⍺ (a and e) , FTH1 (b and e) and eNOS (c). Representative immunoblots are shown below the densitometric analysis of proteins relative to β-actin loading control. Data denote mean ± S.E.M., n = 4 independent cultures. **P<0.05, ***P<0.001*.

**
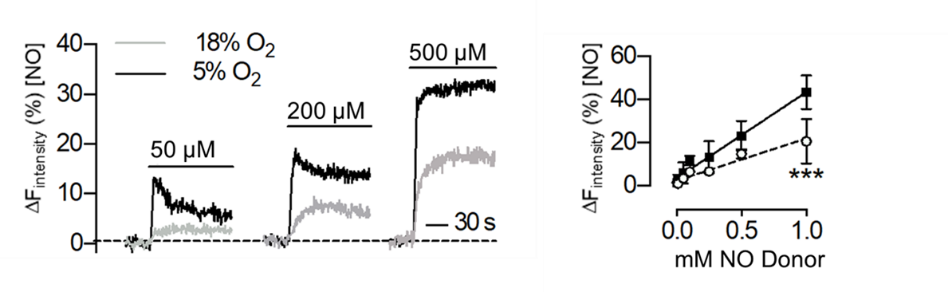
**

**Supplementary Fig. 2: Enhanced intracellular NO bioavailability in HEK293T cells under physiological normoxia.** HEK293T cells stably expressing geNOps were maintained under room air (18 kPa O_2_) or physiological normoxia (5 kPa O_2_) for 5 days and then challenged acutely with NO donor sodium nitroprusside (SNP, 50 – 500 µM) using injector ports in an O_2_-regulated plate reader maintained at 18 kPa or 5 kPa O_2_. Notably, intracellular geNOps signals increased with increasing SNP concentrations under both O_2_ levels, although an enhanced geNOps signal was measured in cells under 5 kPa due to diminished NO scavenging under physiological O_2_ levels. Data denote mean ± S.E.M., n=3, ****P<0.001*.


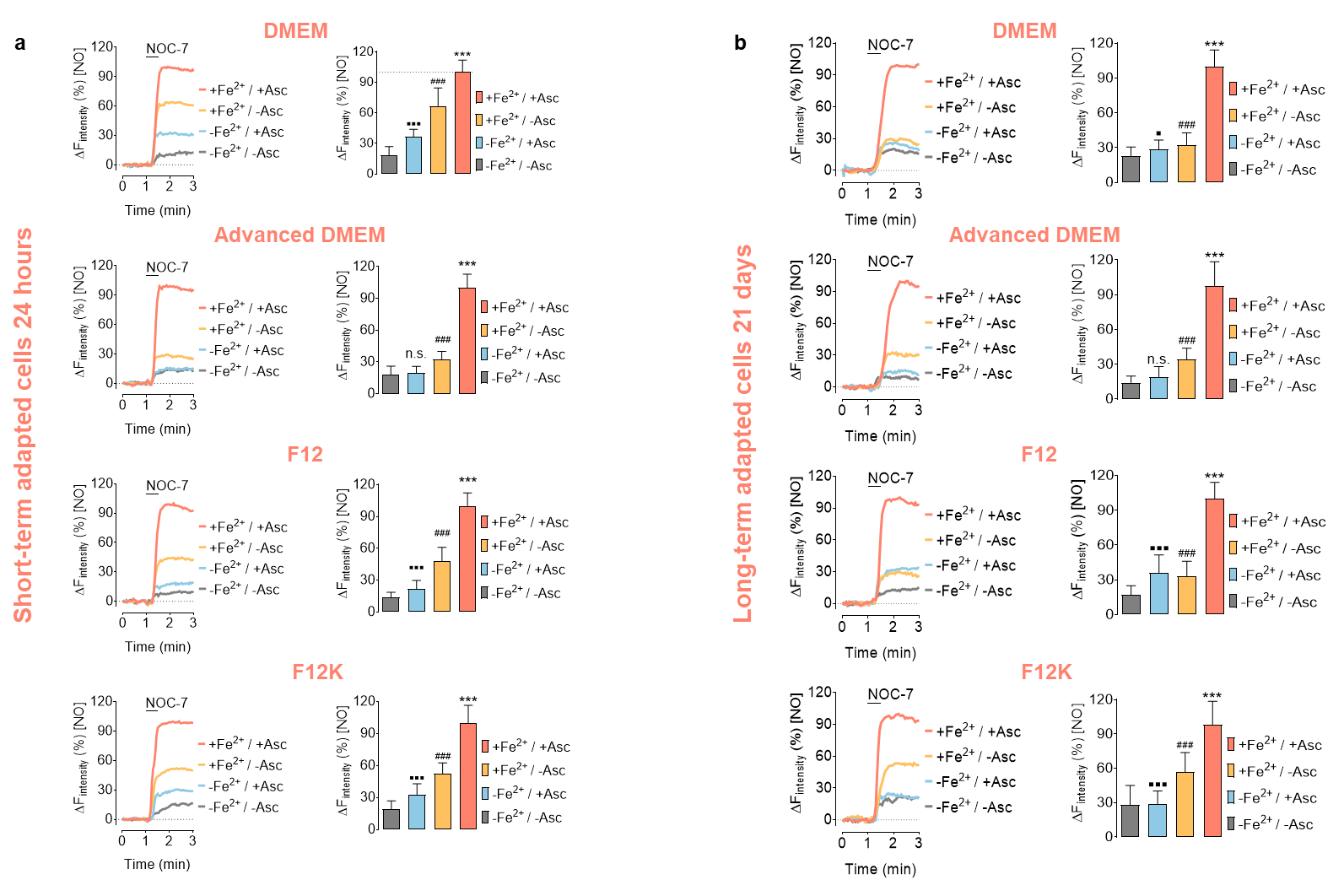


**Supplementary Fig. 3**: **Short or long-term adaptation of HEK293T cells in different culture media under 18 kPa O_2_. a,** Figures in this panel show NO responses of cultured cells in different culture media (DMEM, Advanced DMEM, F12, or F12K) and different iron (II) supplementation. Cells were maintained in the indicated culture media for 24 h. **b,** Figures in this panel show the same procedure as in panel a, but cell treatments were continued for 21 days. After adaptation, either 24 h (a) or 21 days (b) cells were tested for geNOps functionality either without any other iron (II) treatment (light grey curves or bars), or 1 mM ascorbate (blue curves or bars), or 1 mM FeSO_4_ (orange curves or bars), or the combination of 1 mM FeSO_4_ and 1 mM ascorbate (pink curves or bars) for 20 min before imaging. All experiments were repeated in triplicate, and 50-120 individual cells were analyzed for each condition. Dunnett's Multiple Comparison Test was applied to compare all columns with the control column. All values denote mean ± S.D., *P*-value summary: *P<0.0001* (^■■■^Control vs -Fe^2+^/ +Asc; ^###^Control vs +Fe^2+^/ -Asc; ***Control vs +Fe^2+^/ +Asc.


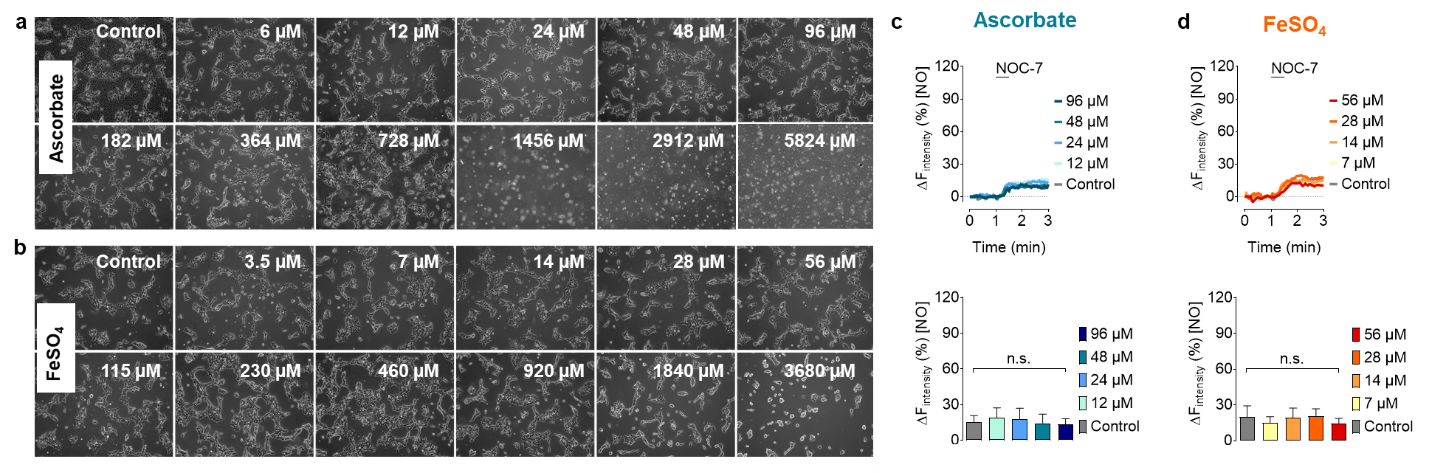


**Supplementary Fig. 4: Long-term adaptation of HEK293T cells to FeSO_4_ or ascorbate under 18 kPa O_2_.** Representative phase-contrast images of cells expressing O-geNOp-NES treated with different concentrations of **a,** ascorbate or **b,** FeSO_4_ as indicated. Representative real-time NO traces in cells expressing O-geNOp-NES adapted for 14 days to increasing concentrations of **c,** ascorbate or **d,** FeSO_4_ as indicated in the legends. All experiments were repeated in triplicates, and 27-111 individual cells were analyzed for each condition. Data denote mean ± S.D., and Dunnett's Multiple Comparison Test was applied to compare all columns to the control column.


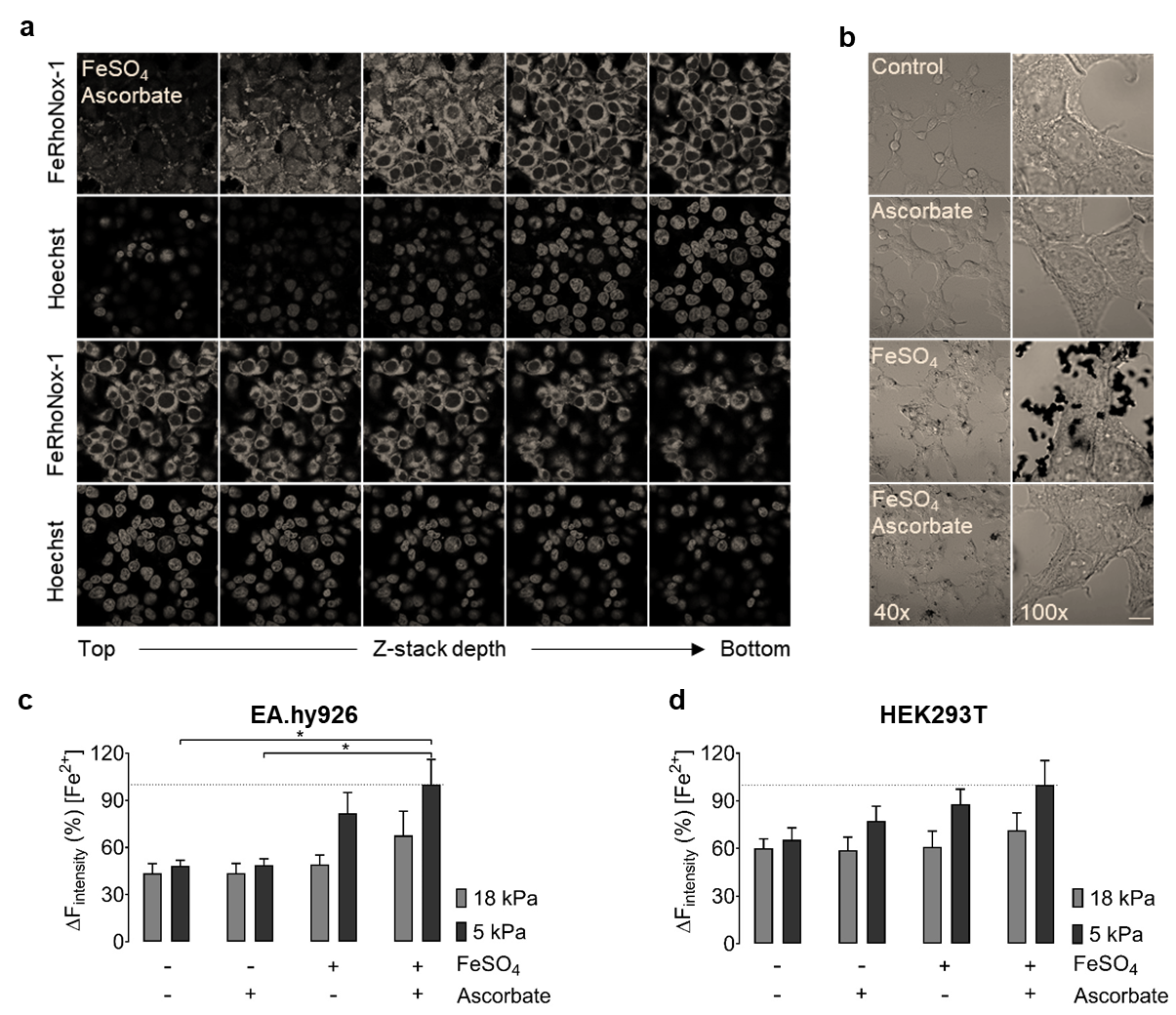


**Supplementary Fig. 5: Live-cell imaging unveils intracellular distribution and iron (II) uptake in EA.hy926 and HEK293T cells following long-term adaptation to 18 or 5 kPa O_2_. a,** High-resolution confocal images show Z-stacks of HEK293T cells treated with 1 mM FeSO_4_ and 1 mM ascorbate for 20 min and co-stained with Hoechst and FeRhoNox-1.  **b,** Phase-contrast images of Perls/DAB stained HEK293T cells under control condition or following treatment with 1 mM ascorbate, or 1 mM FeSO_4_, or 1 mM FeSO_4_ + 1 mM ascorbate for 20 min. Normalized FeRhoNox-1 fluorescent intensities in EA.hy926 **(c)** and HEK293T **(d)** cells following long-term adaption to 18 kPa (light grey bars) or 5 kPa O_2_ (dark grey bars) under non-treated conditions, 1mM FeSO_4,_ 1 mM ascorbate or 1 mM FeSO_4_ + 1 mM ascorbate for 20 min. Data denote mean ± S.E.M., n=6 different cell cultures, Tukey's Multiple Comparison Test, *P*-value summary: **P<0.05.*

**
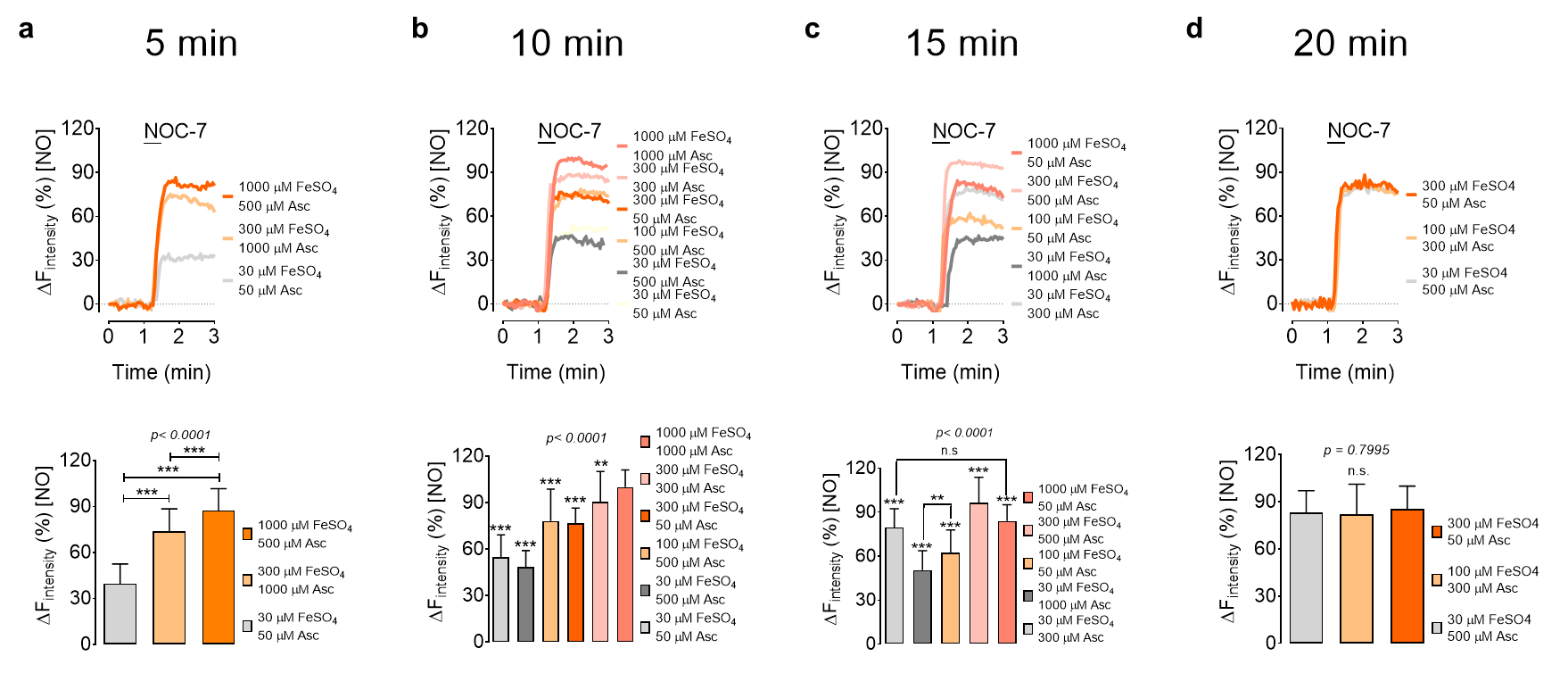
**

**Supplementary Fig. 6: Optimization of iron (II) loading protocol using Taguchi experimental design.** Curves in this figure show real-time traces of NO responses in HEK293T cells under 18 kPa O_2_ and pretreated with different iron(II) and ascorbate concentrations for 5 min **(a),** 10 min **(b)**, 15 min **(c)**, or 20 min **(d)**. Bars denotemean ± S.D. of experiments conducted in duplicate in accordance with Taguchi design of experiments, with the number of individual cells ranging between 25 to 105 cells. For statistical analysis, 1 mM FeSO_4_ + 1 mM ascorbate for 10 min has been used as the control for comparison with other treatments Tukey's Multiple Comparison Test (panel b, right panel), in all other panels, Bonferroni's Multiple Comparison Test was performed. *P-values* are indicated, respectively.


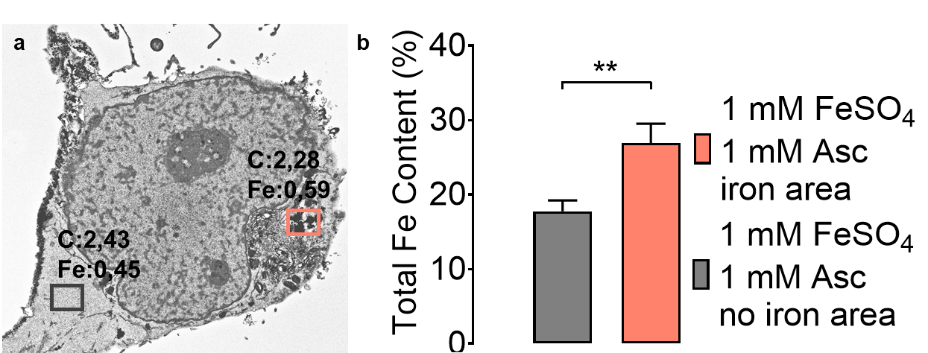


**Supplementary Fig. 7: Scanning electron microscopy with energy-dispersive X-ray spectroscopy (SEM/EDX) to determine total Fe levels. . a,** Representative SEM/EDX micrograph shows the cross-section of an individual HEK293T cell under 18 kPa O_2_ and pretreated with 1 mM FeSO_4_ and 1 mM ascorbate for 20 min. The pink square indicates the accumulation of iron particles. The grey square shows a cell region without iron accumulation. **b,** Bars represent the maximum iron levels in cells detected in iron-containing (pink bar, n=3) and non-iron-containing regions (grey bar, n=3). Total iron levels were normalized against the total carbon content in the respective region of interest. Student's t-test was applied, *P<0.01*.


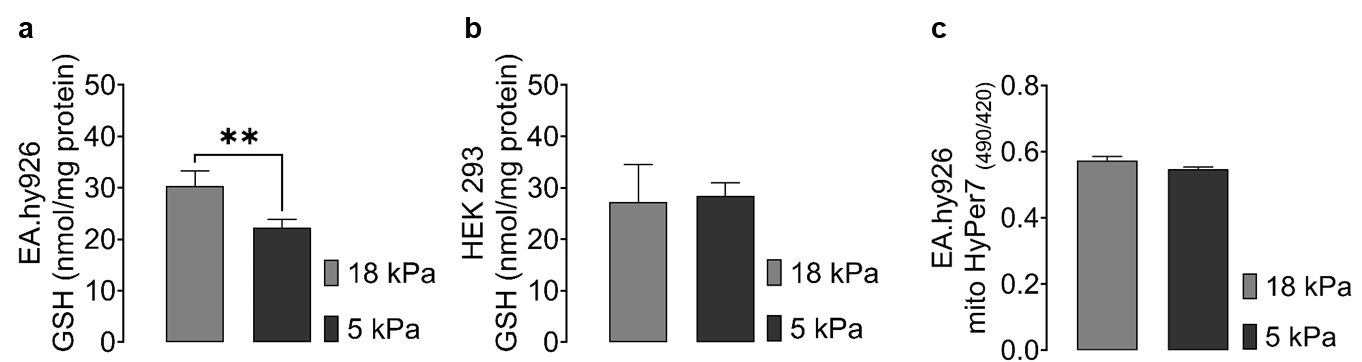


**Supplementary Fig. 8: Basal intracellular GSH and H_2_O_2_ levels in EA.hy926 and HEK293T cells adapted to 18 or 5 kPa O_2_.** Bars show total intracellular glutathione levels in EA.hy926 **(a)** and HEK293T **(b)** cells adapted for 5 days to either 18 kPa (light grey bars) or 5 kPa (dark grey bars) O_2_. **c**, Bars indicate the statistical analysis of basal H_2_O_2_ levels in the mitochondria of EA.hy926 cells adapted for five days to either 18 kPa (light grey bars) or 5 kPa (dark grey bars) O_2_. Data denote mean ± S.E.M., n = 4 independent cultures, Student's unpaired *t*-test, ***P<0.01*.

**Supplementary Table 1**: Experimental design for iron (II) loading optimization


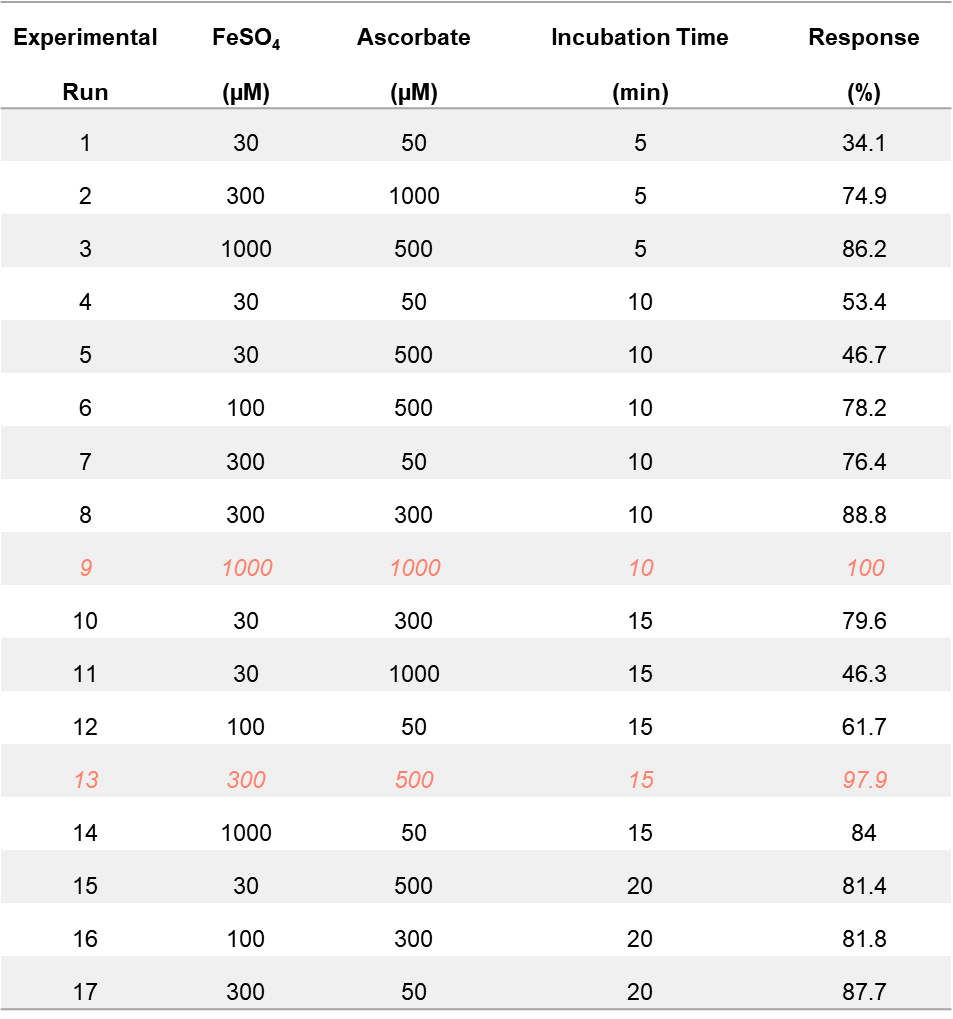

Supplement: Multimedia component 1 [file mmc1.docx]
